# Interleukin-21 Influences Glioblastoma Course: Biological Mechanisms and Therapeutic Potential

**DOI:** 10.3390/cells12182284

**Published:** 2023-09-15

**Authors:** Alberto Repici, Alessio Ardizzone, Alessia Filippone, Cristina Colarossi, Marzia Mare, Gabriele Raciti, Deborah Mannino, Salvatore Cuzzocrea, Irene Paterniti, Emanuela Esposito

**Affiliations:** 1 Department of Chemical, Biological, Pharmaceutical and Environmental Sciences, University of Messina, Viale Ferdinando Stagno d’Alcontres, 98166 Messina, Italy; alberto.repici@studenti.unime.it (A.R.); aleardizzone@unime.it (A.A.); alessia.filippone@unime.it (A.F.); deborah.mannino@unime.it (D.M.); salvator@unime.it (S.C.); eesposito@unime.it (E.E.); 2 Istituto Oncologico del Mediterraneo, Via Penninazzo 7, 95029 Viagrande, Italy; cristina.colarossi@grupposamed.com (C.C.); marzia.mare@grupposamed.com (M.M.); 3 IOM Ricerca, Via Penninazzo 11, 95029 Viagrande, Italy; gabriele.raciti@grupposamed.com; 4 Department of Biomedical, Dental and Morphological and Functional Imaging Sciences, University of Messina, 98122 Messina, Italy

**Keywords:** primary brain tumors, glioblastoma, interleukin-21, cytokines, cancer immunology

## Abstract

Brain tumors represent a heterogeneous group of neoplasms involving the brain or nearby tissues, affecting populations of all ages with a high incidence worldwide. Among the primary brain tumors, the most aggressive and also the most common is glioblastoma (GB), a type of glioma that falls into the category of IV-grade astrocytoma. GB often leads to death within a few months after diagnosis, even if the patient is treated with available therapies; for this reason, it is important to continue to discover new therapeutic approaches to allow for a better survival rate of these patients. Immunotherapy, today, seems to be one of the most innovative types of treatment, based on the ability of the immune system to counteract various pathologies, including cancer. In this context, interleukin 21 (IL-21), a type I cytokine produced by natural killer (NK) cells and CD4^+^ T lymphocytes, appears to be a valid target for new therapies since this cytokine is involved in the activation of innate and adaptive immunity. To match this purpose, our review deeply evaluated how IL-21 could influence the progression of GB, analyzing its main biological processes and mechanisms while evaluating the potential use of the latest available therapies.

## 1. Introduction

Cancer affects millions of individuals worldwide, resulting in it being one of the most prevalent diseases and the second largest cause of death after cardiovascular disease [1].

The common elements among each form of neoplasm include uncontrolled cell development brought on by genetic changes and environmental variables, such as smoking, poor diet, obesity, infections, ionizing radiation, stress, and environmental pollutants [2]. In the setting of the central nervous system (CNS), tumors are unquestionably a deadly illness, representing an important challenge for clinicians, also considering that their incidence has gradually increased in recent years.

In particular, this brain tumor upsurge has become more pronounced in the over-65 age group and greater in males than in women, probably due to a change in lifestyle, especially in industrialized countries [3]. The symptomatology includes headache, nausea, vomiting, mental changes, balance disorders, speech disorders, loss of strength in the limbs, and sensitivity disorders, which typically start slowly and may not be specific.

A multimodal strategy is being used to treat malignant primary brain tumors. The golden approach is a surgical resection, which is followed by radiation and traditional chemotherapy using alkylating drugs such as temozolomide (TMZ).

Nevertheless, the prognosis for malignant primary brain tumors is poor, despite the available pharmaceutical treatments. Indeed, the five-year relative survival rate of primary brain tumors after diagnosis is less than 36%, reaching 6.8% in the case of glioblastoma (GB) [4].

In this context, GB is the most prevalent and deadly subtype of malignant brain tumors. This tumor, also known as a grade IV astrocytoma, is a member of the broad family of gliomas. GB frequently originates from a type of glial cells called astrocytes, and it is characterized by aberrant angiogenesis, apoptotic alteration, and high invasiveness.

The success of therapeutic approaches in glioblastoma is largely restrained by the presence of the blood–brain barrier (BBB). Indeed, the BBB functions as a sort of natural filter to restrict the flow of chemicals, including medications, from the blood to the brain’s tissues, limiting local drug delivery and, consequently, giving rise to therapeutic failure [5].

Thus, bypassing the natural defensive capacity of the BBB, through suitable molecules or carriers, is crucial to developing a type of effective therapy not only for neoplasms but for all diseases that affect the brain.

Nowadays, the understanding of the molecular mechanisms behind GB and the discovery of targeted drug delivery crossing the BBB has generated novel compelling therapeutic approaches, such as immunotherapeutic drugs, thus opening horizons towards new attractive scenarios.

The current immunotherapies involve the use of CAR-T cells, immune checkpoint inhibitors, oncolytic vaccines, and nanoparticles capable of overcoming the biological obstacle of the BBB [6].

Specifically, drugs that target the programmed cell death protein 1 (PD1)—PD1 ligand 1 (PDL1) axis or the cytotoxic T lymphocyte-associated antigen 4 (CTLA4), as well as therapeutic vaccines and chimeric antigen receptor (CAR) T-cell therapy, have undergone preclinical and clinical testing [7].

However, despite the encouraging outcomes of pre-clinical studies, the majority of novel therapies did not demonstrate meaningful benefit in Phase II/III clinical trials [8]. This is probably due to the high intratumor heterogeneity of malignant primary brain tumors and, in certain cases, the inherent limitations of intracranial drug delivery for GB [8].

Interestingly, the latest research focuses on cytokines, proteins, or glycoproteins that function as mediators of intercellular communication to regulate the immune system.

Immune system cells produce cytokines in response to pathogens and tumor antigens, thereby, a variety of cytokines are required for the generation of strong and effective anti-tumor immunity as well as the regulation of immune responses [9].

Thus, it was understood that the development of successful immunotherapeutic strategies to fight cancer depends on the complicated functions of cytokines in delivering the optimum anti-tumor action.

Among interleukins (ILs), interleukin-21 (IL-21) is a recently discovered member of the type-I cytokine family that resembles other ILs, such as IL-7, IL-2, IL-4, and IL-15 [10].

The polypeptide IL-21, which has 131 amino acids and a molecular weight of 15.6 kDa, is pleiotropic and generated by Th1 and Th17 cells, natural killer T (NKT) cells, and follicular helper T cells [11]. Numerous immune system lymphoid and myeloid cells are impacted by IL-21 since this cytokine possesses immunomodulatory capabilities that link most of the immune system components, influencing the development, differentiation, and survival of lymphocytes possessing anti-tumor activity [10].

As a consequence of this potential, IL-21 has drawn major interest to the use of cytokines in brain cancer immunotherapy.

Indeed, it has been reported that in the tumor microenvironment, IL-21 exerts a lot of biological activities, stimulating the Janus family tyrosine kinases (JAK), JAK1 and JAK3, as well as triggering STAT1, STAT3, and STAT5 [12,13,14].

IL-21’s capacity to stimulate and expand cytotoxic CD8^+^ T cells, NK cells, and NKT cells also confers powerful anti-tumor characteristics [15]. In addition to its capacity to inhibit the growth of Foxp3 regulatory T cells (Tregs), IL-21 also affects immunological effectors via modest distance-related connections or even as a membrane-bound isoform [11,16].

Considering these assumptions, it is not surprising that IL-21 has become an important target for the creation of novel treatment strategies to modulate immunity and inflammation in cancer. Thus, this review’s purpose is to assess the molecular processes of IL-21, with a particular emphasis on the therapeutic potential of targeted treatments for the management of glioblastoma.

## 2. Primary Brain Tumors Classification and Features: Focus on GB

An abnormal growth of brain tissue cells leads to the formation of different types of tumors; thus, a tumor that develops directly in the brain or neighboring tissues is considered a primary brain tumor, while a tumor that develops in another location of the body and then spreads in the brain is considered a secondary (or metastatic) brain tumor [17].

A further division into classes is made possible by the main features of the tumor, e.g., benign tumors develop more slowly, while malignant neoplasms grow faster, so much so that they manifest symptoms in the patient very early [18].

The current division of brain tumors is made possible thanks to the 2021 WHO Classification of Tumors of CNS [19]. This document gives us a series of information about the division, genetic differences, new therapies, and diagnoses (Table 1).

Following the guidelines of the WHO, primary head cancers, divided according to the tissue from which they originate, may present with different severity. Glial tumors, originating from glial tissue, are the most common and are divided into four broad categories: low-grade pilocytic astrocytoma (grade I), diffuse astrocytoma (grade II), high-grade anaplastic astrocytoma (grade III), and GB (grade IV).

The growth of neoplasms can lead to symptoms with different manifestations and timing, although certain symptoms are very common, such as headache, weakness, confusion, and seizures [20]. In particular, the development of a tumor mass in the frontal lobe can cause weakness or dysplasia, while if the tumor is located in the parietal lobe, it would lead the patient to problems regarding spatial orientation, thus highlighting how the presence of a neoplasm in different areas of the brain can lead to a different symptomatology [21].

Given the heterogeneity of the clinical picture in patients suffering from brain tumors, some symptoms have been identified that can be considered alarm bells for the diagnosis of brain neoplasms, for example, the sudden appearance of seizures in patients who never had epileptic manifestations [22]. Therefore, the occurrence of these clinical signs or one of the aforementioned symptoms should serve as a prompt for additional in-depth diagnostic investigations.

There are some methods used to diagnose any brain tumor formations including magnetic resonance imaging (MRI) with a contrast agent or computed tomography. These tests, in addition to confirming the presence of a neoplasm, provide a precise indication of the size and location in the various areas of the brain [23]. Moreover, if there is a risk that the tumor has invaded of the surrounding are in the brain, extending to the meninges, a further analysis that can be conducted is the lumbar puncture; in fact, an analysis of the tumor cells present in the cerebrospinal fluid is a useful tool in the definition of patients’ clinical picture. However, a combination of imaging, biopsy, and cerebrospinal fluid analysis is required to confirm the actual characteristics of a tumor [24].

Although the causes of the development of brain tumors are still not clear, age and some genetic predispositions could be considered risk factors. Relatedly, it was estimated that only 5% of gliomas have a hereditary component, and the only known risk factor is exposure to ionizing radiation, while the other environmental factors affecting the development of brain neoplasms are not known [25].

Survival and quality of life are two key objectives to be achieved through the most appropriate therapeutic regimen. From this perspective, patients’ factors, such as age or comorbidity, and aspects that depend on the nature of the neoplasm, such as location and size, are always considered in choosing the best therapy to administer [26].

Nowadays, the treatment for brain tumors involves several approaches. If the tumor is very extensive in a superficial area of the brain, surgery can be attempted. However, surgical techniques have several disadvantages, especially those linked to the position of the neoplasm or its size, since both these factors can make the removal of the tumor mass more difficult, increasing the risk of damage in the internal areas of the brain and neighboring tissues [27]. Current therapies also include radiotherapy and chemotherapy; radiotherapy includes a series of ionizing radiation cycles with the aim of destroying cancer cells [28]; likewise, chemotherapy exploits the cytotoxic power of certain therapeutic agents to kill cells in rapid proliferation [29].

Some of the most commonly used drugs during chemotherapy are temozolomide, nitrosoureas, and organo-platinum complexes [30]. These drugs belong to the alkylating agent’s category, which means they are able to insert an alkyl group into DNA. Their mechanism of action requires that the alkyl groups are inserted between the strands of DNA, preventing their replication and inducing an alteration in the transcription of RNA. Once these mechanisms are blocked, the cells undergo death through an apoptosis process [31].

Furthermore, platinum complexes are also used in therapy as alkylating drugs, exploiting the same concept and intercalating into DNA, acting as a blocker of the growth and replication of cancer cells [32].

Unfortunately, these therapies, alone or in combination, often fail to ensure the total recovery of the patient, only prolonging the patient’s life expectancy. On the other hand, the prolonged use of one or more chemotherapeutics can lead to side effects such as anorexia, diarrhea, headache, hair loss, or myelosuppression [33]. Other common clinical complications after a surgical procedure to treat primary brain tumors include deep vein thrombosis, pulmonary embolism, systemic infections confined to the wound area, seizures, and depression [34].

Despite clinical efforts, the survival rate over 5 years for primary brain tumors such as gliomas is around 44.4% [34,35]. Clearly, survival depends heavily on the specific type of tumor: 100% for pilocytic astrocytoma, 58% for low-grade astrocytoma, 11% for anaplastic astrocytoma, and only 1,2% for glioblastoma [34,35].

Glioblastoma is one of the most aggressive brain tumors; in fact, its prognoses do not ensure 15–16 months of survival after diagnosis, and only 5% of patients have a survival of about 5 years [36]. This tumor also possesses an elevated ability to infiltrate and invade the surrounding tissues, making the surgical approach very challenging. Moreover, the genetic nature of glioblastoma allows it to have a high resistance to therapies (chemoresistance) [37], also increasing the probability of relapses so that 90% of patients will develop a recurrence [38]. It was assumed that tumorigenesis was caused by an increased activation of proto-oncogenes or by the inactivation of genes for tumor suppression. Now, it has been recognized that oncogenesis is not only linked to uncontrolled cell proliferation but also caused by an imperfection in the physiological apoptotic process. Several pathways leading to apoptosis may be compromised in GB cancer cells, such as the PI3K/AKT pathway, which has been reported to be deregulated in 80% of GB [39]. In glioblastoma, pAKT causes an overexpression of the murine protoncogene double minute 2 (MDM2), which negatively regulates the expression of proapoptotic proteins such as p53 [40] and Bad [41].

Moreover, the genetic abnormalities of glioblastoma have been cataloged, according to the Cancer Genome Atlas, into four subtypes: classical, mesenchymal, proneural, and neural [42]. The classical subtype has epidermal growth factor receptor (EGFR) amplification, a loss of tumor protein 53 (*TP53*) and cyclin-dependent kinase inhibitor 2A (*CDKN2A*) genes, and a mutation of the epidermal growth factor receptor variant III (EGFRvIII). The mesenchymal subtype has a mutation of neurofibromin 1 (NF1) and a related absence of phosphoinositide-3-kinase regulatory subunit 1 (PIK3R1), isocitrate dehydrogenase (NADP(+)) 1(IDH1), and platelet-derived growth factor receptor A (PDGFRA), additionally possessing an increased expression of mesenchymal epithelial transition (MET) and chitinase 3 like 1 (CHI3L1), resulting in the activation of the nuclear factor kappa-light-chain-enhancer of the activated B cells (NFκB) pathway [43]. The proneural subtype demonstrates a series of mutations of TP53, IDH1, PDGFRA, and PIK3CA/PIK3R1; Verhaak et al. report that glioblastomas mainly affecting younger patients fall into this category. Finally, there is the last subcategory, which is neural and has increased levels of neurofilament light polypeptide (NEFL) and mutations of phosphatase and the tensin homolog (*PTEN*), *EGFR*, and *TP53* genes [43]. In the genetic context of GB, mutations play a crucial role in the development of high-grade malignancies. A frequent mutation is that of EGFRvIII, which lacks 267 amino acids in the extracellular region, reflected in a constitutionally activated receptor unable to bind to any ligand, thus activating the signal [44]. Another somatic mutation in GB involves the PI3K complex, which includes a catalytically active protein (p110α, encoded by PIK3CA) and a regulatory protein (p85α, encoded by PIK3R) [45]. Missense mutations occur in the adaptor-binding domain (ABD) and in the C2 helical and kinase domains [42]; in-frame deletions in PIK3CA ABD have also been found, which in turn cause a communication disruption between the p110α and p85α subunits [46]. Genetic abnormalities of the IDH1 coding gene are also frequent in glioblastoma; these mutations reduce the affinity of the active site of IDH1 for isocitrate while conversely increasing the affinity for nicotinamide adenine dinucleotide phosphate (NADPH) and α-ketlutogarate (α-KG) [47]. The mutated enzyme subsequently converts α-KG to the R-enantiomer of the metabolite 2-HG (R-2-HG) [47], an oncometabolite that promotes tumorigenesis. The pathways that can be compromised during the process of cell proliferation and differentiation are numerous, each with its own protagonists and implications. For instance, the PI3K/AKT/mTOR signaling pathway has a key role in inhibiting apoptosis during glioblastoma [48]. The activation of PI3K, after a series of phosphorylation leading to the formation of phosphatidylinositol-3,4,5-triphosphate (PIP3), induces the accumulation of a series of signal proteins such as pAkt, which in turn induces an overexpression of MDM2, which is a powerful protooncogene inhibitor of p53 [49]. Akt can also activate mTOR, a kinase protein that may interfere with cell proliferation [50]. Definitely, the PI3K/AKT/mTOR pathway will become a possible pharmaceutical target in the fight against primary brain tumors; countering the abnormal function of one of these factors could lead to a valid therapeutic approach in the glioblastoma contest.

It is therefore evident that the development of a complicated pathology such as glioblastoma can be influenced by many different factors such as the different genetic abnormalities or the various altered pathways mentioned above. Focusing on the phenomenon of cell death will certainly be a key step in the progress of new therapies; it will be essential to perfecting the drugs capable of blocking altered upstream pathways, such as the apoptotic one.

## 3. Role of Immune System in GB: Players and Involved Pathways

The immune system plays a key role in the development of a tumor; indeed, its innate and adaptive response can lead to changes in the tumor microenvironments for each type of neoplasia.

The innate immunity of the brain is guaranteed by the blood–brain barrier (BBB), a functional structure formed by endothelial cells with the characteristic of forming a continuous and non-fenestrated endothelium [51]. Endothelial cells are joined together by tight joints and are wrapped in astrocytic formations that provide additional protection [52].

Thus, the brain and spinal cord are considered ‘immuno-privileged’ organs because of the protection provided by the BBB, which separates them from the other organs, limiting as much as possible the contact with any pathogens [53].

Moreover, the protection of the CNS is guaranteed by the activity of microglia. Microglia cells come from hematopoietic stem cells and play a defensive role by exploiting their high phagocytic capacity [54] and promoting angiogenesis [55]. In addition, these cells can rapidly activate in an ameboid state and release not only reactive oxygen species (ROS) but also pro-inflammatory cytokines [56].

The activation of microglia occurs, as in the immune system, through an interaction between the pattern recognition receptors (PRRs), which recognize pathogen-associated molecular patterns (PAMPs) and damage-associated molecular patterns (DAMPs) [57]. Any insults can also activate the immune system, allowing migration to the central nervous system of macrophages, resulting in the production of different factors such as IL-1, IL-6, or TNF-α, which increase the activation of lymphocytes, proliferation, and the production of effector cells [58]. The mechanisms underlying the release and activation of ILs are multiple and changing. Ils are usually secreted by leukocytes but can also be secreted also by lymphocytes, natural killers, or even endothelial cells [59]. Due to their pro- or anti-inflammatory nature, a response to the pathogens and other antigens is released, modulating and mediating the inflammatory and immune responses, as in the case of allergies [60]. They are cytokines that bind to specific receptors placed on the plasma membrane [61,62]. In this review, we focused on analyzing the role of IL-21 in GB, but there are also other mediators that play an important role in the pathophysiology of this tumor, such as IL-1β. IL-1β is a cytokine that greatly influences the GB pattern, leading to the activation of the key molecules for tumorigenesis [63]. High levels of this interleukin (and its receptor) have been observed in U87GM cell lines [64,65]. The binding to its receptor leads to the activation of NF-κB, P38 MAPK, and JNKs, with an over-regulation of VEGF and consequent angiogenesis, migration, and invasion [66]. Moreover, a secondary effect in GB cells has been seen to be a general worsening of inflammatory conditions with the release of IL-6 and IL-8 and increased levels of cyclooxygenase-2 (COX-2) [67].

The ways in which primary brain tumors evade the control of the immune system are different; the example of glioblastoma and glioma is the most obvious. The immune system finds it difficult to detect cancerous cells because they do not express enough human leukocyte antigen (HLA) [68], an MHC (major histocompatibility complex) class II cell surface receptor. Moreover, the ability to evade the surveillance of the immune system resides within the tumor microenvironment species; some of the factors that can be secreted are TGF-β, IL-4, and IL-10 [69].

These particular cytokines regulate transcription factors such as the signal transducer and transcription activator 3 (STAT3) in tumor cells, especially in gliomas, resulting in tumor-causing immune responses and also leading to an inhibition of T cell activation and proliferation [70]. Among the different mediators involved in the tumor microenvironment, TGF-β is perhaps the most critical factor in proliferation, invasion, immunosuppression, and angiogenesis. Human studies have also shown that TGF-β is overexpressed in tissues from gliomas but not detectable in healthy brain tissues [71], so in glioblastoma, the TGF-β2 isoform is particularly altered [72].

The expression and activation of certain receptors lead, in some primary tumors of the central nervous system such as glioma, to a reduction in the activity of natural killer (NK) cells, cytotoxic T lymphocytes (CTL), and the decreased proliferation of T cells; in this regard, the receptors most involved are HLA-G, HLA-E, and prostaglandin E receptor 2 (PTGER2) [73]. Human leukocyte G and E antigens (HLA-G and HLA-E) are the non-classical class I major histocompatibility complexes involved in immune system regulation. It has been shown that HLA-G is associated with the ability of tumors (even in GB) to evade the immune system, and because it is overregulated, it could become a valid therapeutic agent [74]. HLA-E, on the other hand, plays an important role in the recognition of NK cells at the cellular level. HLA-E binds to several peptides and is then expressed at the cell surface. NK cells recognize the HLA-E+peptide complex via CD94/NKG2A receptors, finally producing an inhibitory effect on cytotoxic activity, preventing lysis [75,76]. Payner and colleagues also highlighted that an increased expression of E2 prostaglandin (PGE2) is associated with growth stimulation in the U87-MG human glioma cell line, underlining that PGE2 mediates GB cell activities, at least in part, through the cAMP/PKA pathway [77].

Then, there are two other factors that intervene during the phenomena of immunity suppression in brain tumors. Indeed, the *CD274* and *FASLG* genes codes, respectively, are influenced by programmed ligand-death 1 (PDL-1) and FasL; these two transmembrane proteins further inhibit the activation of T cells. Programmed ligand-death 1 (PDL-1) is a type-1 transmembrane protein that interacts with its PD-1 receptor by inactivating T cells and causing immune tolerance [78]. FasL, on the other hand, a type-II transmembrane protein expressed on cytotoxic T cells and NK cells, belongs to the tumor necrosis factors (TNF), and its activation leads to cellular apoptosis, while its downregulation is exploited by cancer cells to survive and escape the surveillance of the immune system [79].

ILs are the mediators that are actively involved in the immune system’s response. These cytokines are usually produced by cells of the immune system (lymphocytes, NK cells, phagocytes, and dendritic cells) following exposure to antigens or pathogens [80]. There are different types of IL-s, each performing a different task; in fact, depending on the IL of interest, these molecules have the ability to be anti-inflammatory (IL-4 and IL-10) and pro-inflammatory (IL-2 and Il-6) [81]. The anti-inflammatory effects of IL-4 are performed through a reduction in the levels of TNF, IL-1, and PGE2, modulating both the inflammatory and immune reactions [82]. On the other hand, IL-10 binds to its receptor, activating JAK1 and non-receptor protein kinase tyrosine (TYK2) and resulting in the activation of STAT3, which promotes the anti-inflammatory action [83]. Cases of neurodegeneration have also been noted in which IL-10 inhibited apoptosis via the pathway of PI3K-Akt, reducing the expression of the anti-apoptotic factor Bcl-2 while attenuating the levels of the pro-apoptotic factor caspase-3 [84]. Of the mentioned pro-inflammatory interleukins, it is possible to consider IL-2, which is produced by CD4^+^- and CD8^+^-activated T cells, as an IL involved in the processes of regulatory T cells and also in the homeostasis of the immune response [84]. In the course of glioblastoma, IL-6 is also involved, in fact, it possesses an anti-inflammatory action and influences cell invasion and migration [85].

Finally, this interleukin affects the tumor microenvironment by activating the JAK/STAT pathway. Once this signaling pathway is active, the factors involved in tumor angiogenesis, such as NF-κβ, TNF-α, MMP-9, MMP-2, and VEGF, are overexpressed [86].

In tumors, the role of ILs is of fundamental importance for communication between cancer cells and the immune system. Several signaling pathways involve IL regulation, spreading an inflammatory state, or impaired immune activation, which significantly contribute to tumor growth, proliferation, and differentiation [87]. Here, we focused on IL-21’s features.

## 4. Role of IL-21 in GB: Significance and Effectiveness of Immunotherapy

IL-21 is a type-1 immunomodulating pleiotropic cytokine coded by the *IL-21* gene on chromosome 4, and it is produced by NK cells and CD4^+^ T lymphocytes and has effects on the innate and adaptive immune systems. The IL-21 receptor, IL-21R, is provided with the γc chain, shared with IL-2, IL-4, IL-7, IL-9, and IL-15 receptors, and a unique receptor [88], expressed by the B and T lymphocytes and NK [89]. A possible mutation of γc leads to X-linked severe combined immunodeficiency (X-SCID), an immunodeficiency disorder in which the body produces too few T cells and NK cells and in which also B cells are defective [90]. This cytokine is pivotal since it play different effects in different cell types such as CD4^+^ and CD8^+^ B cells, T cells, monocytes, macrophages, and dendritic cells (DCs) [91].

When the IL-21 binding occurs, it can trigger a cascade mechanism analogous to that of class I cytokines, with an effect achieved through the Janus kinase and signal transducer and activator of transcription (Jak-STAT) pathway [92].

**Figure 1:**
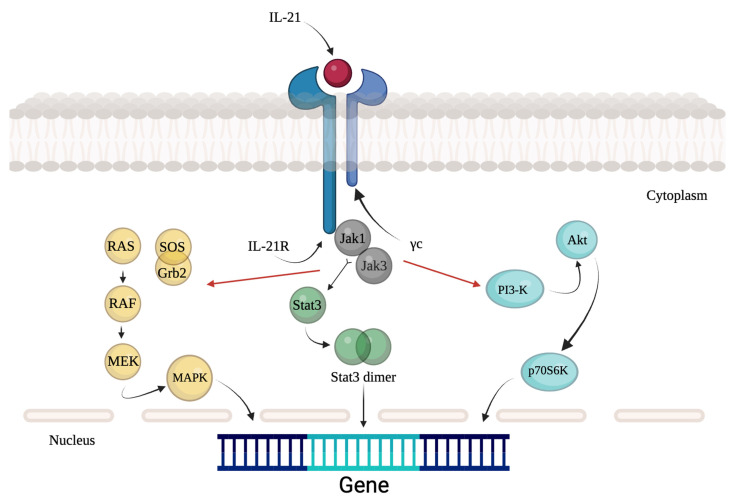
Signaling pathway of IL-21.

The first activated mediators are JAK1 and JAK3, which fall into the group of Janus family tyrosine kinases, which in turn activate STAT1 and STAT3 [93] (as illustrated in Figure 1).

Moreover, the activation of IL-21R can lead to the activation of other pathways, such as those of the MAP kinase (MAPK) or phosphoinositide 3-kinase (PI3K) [93].

As reported by Spolski et al., IL-21 can have multiple effects; the most curious one is the proapoptotic effect on the B lymphocytes, depending on the context [94]. It has been shown that the phenomenon of apoptosis prevails when B cells have been activated by a Toll-like receptor (TLR) as LPS; conversely, this IL can increase the proliferation of B cells when these are activated by a B-cell receptor signal (BCR) in addition to co-stimulator signals such as those provided by anti-CD40 [94].

It is important to point out that the pro-apoptotic activity of IL-21 is caspase-dependent as a consequence of the induction of the B cell lymphoma 2 (BCL-2)-interacting mediator of cell death (BIM), a pro-apoptotic protein [94]. Beyond the pro-apoptotic effects, it has been observed that IL-21, in combination with the monoclonal antibodies anti-CD20 rituximab and fludarabine, can indirectly kill cells now insensitive to the effects of IL-21 by enhancing the cytotoxic power of the NK cells. In addition to its pro-apoptotic power, treatment with IL-21 has enhanced the cytotoxic activity of the two chemotherapeutics mentioned before, also promoting the phosphorylation of STAT1 and STAT5 in NK cells against rituximab-coated chronic lymphocytic leukemia cells in vitro [95].

Another effect that IL-21 may have is to regulate the production of immunoglobulins by B lymphocytes [96].

Although it is known that the role of IL-21 is to reduce the expression of IgE, it also promotes the expression of IgG during the response of hypersensitivity type I, helping to mitigate them [97]. In mice with IL-21R knockout (KO), there was a general reduction in the immunoglobulin response (IgG1) and an increased production of IgE after an antigen exposure [97].

Clearly, given the role that IL-21 plays in mediating both types of immunity, any imbalance in its regulation (and in its receptor) could lead to autoimmune diseases, as in the case of animal models with type 1 diabetes [98] or systemic lupus erythematosus [99].

It was noted that in pathologies such as lupus [100] or rheumatoid arthritis [101], the number of CD4^+^ T lymphocytes producing IL-21 increased, suggesting that blocking this IL could improve the conditions of these pathologies.

Boosting immune system responses is the basis of immunotherapy, and in this appealing field, IL-21 appears to be a valuable therapeutic agent. In fact, this cytokine not only enhances cytotoxicity and promotes maturation but also manages the production of IFN-γ and perforin from NK cells [102], also contributing together with IL-15 to increase the number of CD8^+^ T lymphocytes, thus favoring effector function and promoting tumor regression [103].

Moreover, IL-21 is able to intervene during chronic viral infections, proving to modulate CD4+ and CD8^+^ lymphocytes, which lose effectiveness in these conditions. In this regard, Elsaesser and colleagues demonstrated, in a mouse model of chronic infection, that IL-21 is not only indispensable for CD4^+^ T lymphocytes by also validating that in the absence of the IL-21 signaling pathway, a CD8^+^ T lymphocyte alteration occurs [104]. Furthermore, another study in which mice had deficiencies of IL-21 or IL-21R showed decreased clonal excretion of CD8^+^ T cells and persistent viral titers, further confirming the relationship between the role of IL-21 and immunity [105].

Hence, it is obvious that IL-21 affects the oncogenic course by managing and redefining the tumor microenvironment through the manipulation of immune-inflammatory pathways given its capacity to affect cell proliferation, immunity, and other crucial biological processes.

Despite the partial knowledge of IL-21’s abilities in tumor microenvironment, the use of IL-21 as a real medicine is currently not applied. The ability of IL-21 to influence the immune system can certainly be used to combat certain types of cancer, such as glioblastoma; however, few studies have evaluated the application of IL-21. Moreover, despite the developments in the use of IL-21 in immunotherapy, the potential to control and counteract metastases is still under study. In theory, given the influence that IL-21 exerts on the immune system, especially NK cells and T cells, metastases should be effectively countered. Sun and colleagues studied an IL-21 vaccine virus, in combination with checkpoint inhibitors, in the treatment of glioma [106]. Theirs was a mouse model in which the vaccine was administered both intrathecal and intravenous, enhanced thanks to treatment with checkpoint inhibitors [106]. The two treatments had a synergistic effect through being able to evoke anti-tumor immunity, thus influencing the tumor microenvironment [106]. Tumor growth inhibition has also been demonstrated in C57BL/6 mice treated for 6 days with the intrathecal administration of a vaccine virus (VV) expressing IL-21 called VVLΔTK-STCΔN1L-mil-2. In addition, its combination with α-PD1 showed a strong therapeutic effect, with an 80% percentage of healing and no recurrence in the next observation time of 180 days. Through a cytofluorometric analysis of the splenocytes, they also analyzed the activity of T cells, dendritic cells (DC), and macrophages; the number of CD4^+^ and CD8^+^ found in the spleen had significantly increased. Dendritic cells have also been markedly activated following an infection with this recombinant virus. Even the presence of this virus inside the cancer cells has produced a beneficial effect because a series of dangerous signals have been activated, resulting in a massive infiltration of immune cells in the tumor microenvironment. Another attempt to enhance the capabilities of the immune system occurred thanks to Tran and colleagues [107]. They tried to enhance the effect of NK cells during treatment with bevacizumab and irinotecan in a mouse model of glioblastoma [107]. The strengthening of the immune system, in this study, took place through the use of natural killer cells, expanded and activated using K562 cells, which express the ligand OX40 and IL-18 and IL-21 linked to the membrane [107]. In their work, K562-OX40L-mb-IL-18/IL-21 showed cytotoxic effects against glioblastoma only in vitro but did not show therapeutic effects in U87 glioblastoma models. The anti-cancers used during this experiment have both consolidated their cytotoxic effect and slowed the development and growth of the tumor in the early stages [107]. In this study, an attempt was made to exploit feeder cells (K562-OX40L-mb-IL-18/IL-21), a layer of cells unable to divide but which provide extracellular secretions to help other cells proliferate, to enhance the immune action of adoptive NK cells and subsequently be used in the treatment of U87 in both in vivo and in vitro models of GB. This study did not report the expected results but emphasized the cytotoxic activity of complexes with bevacizumab and irinotecan. IL-21 was therefore used as an adjuvant. IL-21, like many other ILs, undergoes an alteration during cancer, not only in the brain. Bender and colleagues compared two different immunoassay models fluorescence, Luminex (LMX) and Meso Scale Discovery (MSD), to evaluate plasma cytokines in patients with glioblastoma, melanoma, lung, or breast cancer metastases to the brain compared with healthy patients [108]. Bender and colleagues produced an interesting study to analyze the circulating biomarkers during GB progression. They compared 19 concentrations of cytokines (including IL-21) in the plasma of newly diagnosed glioblastoma patients and healthy patients using two different multiple immunoassay platforms. Both methodologies confirmed that IL-21, along with other cytokines, was overexpressed in the samples from patients with GB compared to the samples from healthy volunteers. It should be noted that during the analysis of IL-21, although the minimum limit of quantification of MSD was 0.22 pg/mL, it was not possible to detect any value. As regards LMX, it instead identified IL-21 in all samples with a range of 1.8/18.9 pg/mL.

Liu et al. demonstrated for the first time the immunological importance of mesothelin as a potential target, using a combination of the gamma chain cytokines IL-2, IL-15, and IL-21 [109]. Mesothelin is a 40 kDa tumor differentiation antigen normally expressed on mesothelial cells but overexpressed in some tumors [109].

Liu et al. found that T cells cultured in the presence of some interleukins, including IL-21, showed enhanced proliferation of mesothelin-specific CD4^+^ and CD8^+^ by showing that the immune cells of patients with GB can recognize and respond strongly to mature mesothelin, bound to the cell surface by glycoinositol-phoslipid (GPI), through increased cytokine production (IFN-γ, TNF-α). Furthermore, the T cells of patients with GB are able to expand greatly in the presence of a medium containing a cocktail of IL-2/IL-15/IL-21 [86]. That is why IL-21, along with IL-2, IL-15 and mesothelin peptides, have been employed to evaluate humoral response using blood samples from patients with glioblastoma [109]. Mesothelin was able to induce a powerful humoral and cellular response, which was also detected at the level of systemic circulation [109].

Assuming that these three ILs produced an effective immune response, in the following study, Liu et al. [110] determined that NY-ESO-1 or survivin expression could be viable targets for anticancer-directed T-cell therapy. Liu et al. used a set of interleukins, including IL-21, to evaluate the expression of two proteins: survivin (*n* = 40 samples) and NY-ESO-1 (*n* = 38 samples). They observed the strong reactivity of T cells using IL-2/IL-15/IL-2, given by the production of IFN-γ in the blood of patients with grade III glioma compared to patients with grade II glioma. Thus, the use of IL-21 in conjunction with IL-15 could prevent the apoptosis of T cells. Since there is currently no optimal therapy against GB possessing no side effects, the possibility of using T cells directly targeting NY-ESO-1 and taken from the peripheral blood mononuclear cells of healthy patients could represent an innovative approach to GB therapy [110]. New approaches to cancer therapy will be needed in the future; an innovative approach has certainly been that of Moyes et colleagues [111]. In fact, the goal of their work was to minimize the immunosuppressive environment of GB by exploiting a genetically engineered macrophage-based platform with the ability to improve the innate and adaptive immune responses. These engineered macrophages possess the versatility to secrete both factors that activate the immune response, such as IL-21, and factors that induce immune suppression. In this way, the primary human macrophages were modified by a lentivirus to make them clinically ideal and useful. Here, after the GB cell U87 injection intracranially for 7 days and LPS/IFN stimulation, IL-21 appeared to prevent the IL-10 expression induced by LPS and also to reduce the expression of IL-10 by 76.4%, showing that in cooperation between engineered macrophages, IL-21 improved the microenvironment features of GB.

Immunotherapy, as a valuable approach against cancer, was further confirmed by other findings; in fact, Wölfl and colleagues showed that it is possible to provoke a widespread expansion of antigen-specific T cells using purified naïve CD8^+^ T cells in combination with ILs such as IL-21, IL-7, and IL-15 in human glioblastoma samples [112]. The work of Wölfl and colleagues has provided us with the possibility of obtaining tumor antigen-specific T cells through a short but intensive ex vivo expansion. The main antigen taken into consideration was Melan-A (also known as MART1), classified among the top 20 tumor antigens fundamental for developing immunotherapy in different types of cancer. By using highly purified naïve CD8^+^ T cells, a single stimulation was performed with mature dendritic cells and antigen-specific peptides. This stimulation was followed by the sequential use of three cytokines: IL-21, IL-7, and IL-15. The results showed the extensive expansion of antigen-specific T cells with tumor-reactive activity, multifunctionality, and the maintenance of a phenotype similar to central memory.

Also stimulating Vγ9Vδ2 T lymphocytes, the main peripheral γδ T cell, via IL-21, is effective in eliminating glioblastoma cells in an in vivo orthotopic model [113]. In this work, the role of allogeneic human TγδVγ9Vδ2 against GB was deepened. The effects of the ex vivo sensitization of T cells Vγ9Vδ2 by IL-21, an immunostimulant cytokine, on their cytolytic reactivity were analyzed. What emerged was that primary human GB cells were naturally eliminated by allogenic TγδVγ9Vδ2 cell, through cytotoxicity mediated by enzymes such as perforin and granzymes. IL-21 thus increased both the intracellular expression of granzyme B and cytotoxicity of human allogeneic TγδVγ9Vδ2 lymphocytes in vitro. Directly stimulating these lymphocytes allowed them to directly increase their cytotoxicity, bypassing the side effects of the direct administration of this cytokine.

Starting from the concept that immunotherapy is certainly one of the last frontiers in the treatment of cancer, in the work of Daga et al. [114], a mouse orthotopic model of glioma was studied using either genetically modified IL-21 or recombinant IL-21 cells as the treatment. What has been noted is that the local injections of IL-21 (or recombinant) induced a significant antitumor response, enhancing the response of NK cells and immune system cells. Daga et al. developed a system that harnessed the ability to massively stimulate the immune system by exploiting IL-21. A mouse orthotopic model with slow-growing GL261 (GL D2-60) cells was used. To surmount the poor immunogenicity that the glioma cells injected intracranially possessed, the same neoplastic cells were transduced with retroviruses-containing genes for the cytokines of interest provide the appropriate volume of cytokines directly at the site of interest. In addition to IL-12, IL-2 was also used for its similar immune characteristics against GB cells. The combination of IL-2 and IL-12 showed beneficial additive effects, resulting in the survival of 55% of the animals after primary implantation and an overall survival of 50% after the second treatment. According to this work, IL-21 has proven significantly better than IL-2 at inducing long-lasting immunity without producing major side effects at the level of the brain.

Furthermore, the expression of IL-21, along with other cytokines, has been considered a marker for predicting the survival of a patient with glioblastoma. In the work of Zhenjiang and colleagues [115], the peripheral blood of patients with glioblastoma was analyzed (glioblastoma = 145; non-glioblastoma = 60) to measure the circulating T cells in the absence or presence of serum cytokines such as IL-2/IL-15/IL-21. Factors such as IL-4/IL-5/IL-6 or IFN-γ/TNF-α/IL-17A in the serum, detectable prior to surgery or GB therapy, may predict the survival of glioblastoma patients. Furthermore, the stimulation of IFN-γ, always through this cocktail of cytokines, can happen from the specific circulating T lymphocytes for EBV, CMV, or the peptide survivin97-111. The results obtained show that about 2% of patients with GB and 18% of patients with non-GB gliomas were still alive 1000 days after surgery or therapy; these patients had a combination of cytokines (IL-4/IL-5/IL-6, IFN-γ/TNF-α/IL-17A). The following were considered as independent survival factors: IL-2/IL-15/IL-21 in conjunction with serum IFN-γ/TNF-α/IL-17a.

Although IL-21 represents an ideal therapeutic approach to activating and boosting the immune system, there are currently no real treatments based its use; indeed, all the studies conducted so far have not had IL-21 as a protagonist but only as an adjuvant therapeutic agent. All the studies presented in this section are summarized in Table 2.

## 5. Conclusions and Future Perspectives

To improve the effectiveness of anticancer therapy, a number of therapeutic approaches have been developed to modify the immune system. In this research area, IL-21 has been and continues to be intensively studied and many of its real applications are not entirely clear. New insights about its use are yet to be clarified because of the IL-21 potential therapeutic use. Furthermore, exploiting murine models, or even better, performing clinical trials will be certainly excellent starting points for evaluating the critical role of IL-21. By using IL-21, GB therapy would represent a totally innovative approach from an anticancer perspective since the activation and enhancement of the immune system is a key step to counteracting tumors, especially in those types that are extremely affected by a highly altered microenvironment, such as brain tumors. As a result, it is expected that more IL-21-targeting therapeutic compounds will be created in the future, providing an innovative overview in the fight against primary brain tumors such as glioblastoma and boosting patients’ chances of survival.

## Figures and Tables

**Table 1:** 2021 World Health Organization Classification of Tumors of the CNS.

Gliomas, Glioneuronal Tumors and Neuronal Tumors	Choroid Plexus Tumors	Embryonal Tumors	Pineal Tumors	Mesenchymal, Non-Meningothelial Tumors	Tumors of the Sellar Region
Adult-type diffuse gliomas		Medulloblastoma		Uncertain differentiation	
Pediatric type diffuses low-grade gliomas		Other Central Nervoys System embryonal tumors			
Pediatric type diffuses high-grade gliomas					
Circumscribed astrocytic gliomas					
Glioneuronal and neuronal tumors					
Ependymal tumors					

**Table 2:** Studies involving IL-21 in the GB.

Cancer Type	Therapeutic Target	Conclusions	Reference
Glioblastoma	Use of a vaccinia virus to treat a mouse model of GBM by releasing IL-21.	Treatment in combination with checkpoint inhibitors has significantly improved the condition of the GB.	[106]
Glioblastoma	NK expansion and increase with K562 cells expressing the ligand OX40 and IL-18 and IL-21.	It shows the limited therapeutic potential of therapy with irinotecan and bevacizumab to treat GB.	[107]
Glioblastoma (also other types)	Evaluation with two different methods to evaluate circulating cytokines in patients with GB.	Double analysis of serum levels of different ILs via LMX and MSD.	[108]
Glioblastoma	Enhance the immunological activity of mesothelin through the following gamma chain cytokines: IL-2, IL-15, and IL-21.	The immunological importance of mesothelin via IL-2, IL-15, and IL-21.	[109]
Glioblastoma	T cell stimulation via a cocktail of ILs: IL-2, IL-15, and IL-21.	The presence of NY-ESO-1 or survivin in GB is a valuable target for cancer-directed T cells.	[110]
Glioblastoma	Genetically engineered macrophages capable of promoting the activation of other cells via IL-21.	Genetically engineered macrophages are an ideal cell for remodeling the tumor microenvironment and enhancing antitumor immunity.	[111]
Glioblastoma	CD8^+^ T cell stimulation with three different ILs, including IL-21.	Use in immunotherapy of multifunctional T cells to counteract different malignancies.	[112]
Glioblastoma	A particular type of T cells, Vγ9Vδ2, stimulated with IL-21.	T cells Vγ9Vδ2 stimulated with IL-21 significantly eliminated GB cells.	[113]
Glioblastoma (also other types)	Exploit the modified *IL-21* gene or recombined IL-21 for immunotherapy.	Local treatment of IL-21 may be appropriate to treat GB.	[114]
Glioblastoma	The study of the patterns of some cytokines with the survivin.	The relationship between some serum cytokines and lymphocytes with survivin_97-111_ is deepened to predict the survival of patients with GB.	[115]

## Data Availability

Not applicable.
